# Alternative divalent cations (Zn^2+^, Co^2+^, and Mn^2+^) are not mutagenic at conditions optimal for HIV-1 reverse transcriptase activity

**DOI:** 10.1186/s12858-015-0041-x

**Published:** 2015-05-03

**Authors:** Vasudevan Achuthan, Jeffrey J DeStefano

**Affiliations:** Department of Cell Biology and Molecular Genetics, University of Maryland, College Park, MD 20742 USA

## Abstract

**Background:**

Fidelity of DNA polymerases can be influenced by cation co-factors. Physiologically, Mg^2+^ is used as a co-factor by HIV reverse transcriptase (RT) to perform catalysis; however, alternative cations including Mn^2+^, Co^2+^, and Zn^2+^ can also support catalysis. Although Zn^2+^ supports DNA synthesis, it inhibits HIV RT by significantly modifying RT catalysis. Zn^2+^ is currently being investigated as a component of novel treatment options against HIV and we wanted to investigate the fidelity of RT with Zn^2+^.

**Methods:**

We used PCR-based and plasmid-based alpha complementation assays as well as steady-state misinsertion and misincorporation assays to examine the fidelity of RT with Mn^2+^, Co^2+^, and Zn^2+^.

**Results:**

The fidelity of DNA synthesis by HIV-1 RT was approximately 2.5 fold greater in Zn^2+^ when compared to Mg^2+^ at cation conditions optimized for nucleotide catalysis. Consistent with this, RT extended primers with mismatched 3′ nucleotides poorly and inserted incorrect nucleotides less efficiently using Zn^2+^ than Mg^2+^. In agreement with previous literature, we observed that Mn^2+^ and Co^2+^ dramatically decreased the fidelity of RT at highly elevated concentrations (6 mM). However, surprisingly, the fidelity of HIV RT with Mn^2+^ and Co^2+^ remained similar to Mg^2+^ at lower concentrations that are optimal for catalysis.

**Conclusion:**

This study shows that Zn^2+^, at optimal extension conditions, increases the fidelity of HIV-1 RT and challenges the notion that alternative cations capable of supporting polymerase catalysis are inherently mutagenic.

## Background

Divalent cations are essential co-factors for polymerase catalysis and are also required for the RNase H activity of reverse transcriptase (RT) [1,2]. HIV-1 RT is a heterodimer consisting of p66 and p51 subunits, with the p66 subunit performing both the polymerase and RNase H activities [3]. Under physiological conditions, Mg^2+^ functions as the co-factor for both activities. In addition to Mg^2+^, RT *in vitro* can use alternative divalent cations such as Mn^2+^, Cu^2+^, Co^2+^ and Zn^2+^ for polymerase activity [4]. These cations are important to many cellular processes and are tightly regulated. The total concentration of Zn^2+^ in cells is ~0.1-0.5 mM [5-8] while the total concentration of Mn^2+^ in red blood cells is ~2.5- 3 μM [9,10], and Co^2+^ in the serum is in the low μM range [11]. The available free concentration of all these cations is kept extremely low by cellular mechanisms [12,13]. Therefore, we believe these divalent cations do not play a significant role in the HIV replication lifecycle.

However, Zn^2+^ is a potent inhibitor of several viral polymerases [14-18] and Zn^2+^, in addition to Mn^2+^, has been shown to inhibit Mg^2+^-dependent HIV RT activity *in vitro* in work from our lab and others [4,19-21]. Other groups have demonstrated that Zn^2+^-based drugs can inhibit HIV spread in animal models [22-27]. Zn^2+^ is an active ingredient of topical solutions under study for the treatment of HIV [25,26] and herpes simplex, an infection that can increase HIV transmission [28-33]. Zn^2+^ has been investigated in several past and current HIV therapeutic trials [34], and is a proposed treatment for rhinovirus infections [35,36]. Therefore, understanding how Zn^2+^ and other divalent cations affect different properties of RT is potentially important for future drug development.

One of the most notable effects of alternative divalent cations on polymerases is alteration of polymerase fidelity. Mn^2+^, Co^2+^, and Ni^2+^ have all been shown to dramatically decrease the fidelity of DNA synthesis by several human, bacterial, and viral polymerases including HIV RT [37-43]. Mn^2+^ and Co^2+^ decreased the fidelity of avian myeoblastosis virus (AMV) RT and human DNA polymerase I in a concentration-dependent manner [40]. Increased error frequency in presence of Mn^2+^ has also been observed *in vitro* with HIV RT [43], *Escherichia coli* DNA polymerase I [44], phage T4 DNA polymerase [45], DNA polymerases α and β [46], and *Taq* polymerase [47]. Most of these experiments were performed using concentrations of divalent cation higher than those required for maximal enzyme activity. However, we recently reported that physiological Mg^2+^ concentrations, which are lower than the high concentration typically used to optimize enzyme kinetics *in vitro*, can increase RT fidelity [48].

Given the potential of Zn^2+^-based compounds as novel drugs against HIV and the vast amount of literature on alternative cations like Mn^2+^ and Co^2+^ being pro-mutagenic at elevated concentrations, we wanted to investigate the fidelity of HIV RT with each of these cations. Although Mn^2+^ and Co^2+^ were previously demonstrated to support RT catalysis, our recent publication [20] was the first to show (to our knowledge) that Zn^2+^, a potent polymerase inhibitor, can also support polymerase catalysis [15]. Therefore, we wanted to look more closely at how this previously untested divalent cation affects RT fidelity. A better understanding of the fidelity of RT with these alternative cations could also be important for modulating the accuracy of RT-PCR reactions. Mn^2+^ is already being used in PCR reactions to generate random mutations [47]. In this report, we show that under optimal extension conditions, Zn^2+^ increases the fidelity of RT, a previously unprecedented observation of an alternative cation for a polymerase. We also show that presumed pro-mutagenic cations, such as Mn^2+^ and Co^2+^, are not mutagenic with HIV RT at concentrations optimal for dNTP catalysis. The potential mechanisms by which Zn^2+^ enhance fidelity as well as the reason for the concentration-dependence of mutagenesis is discussed.

## Results

### Estimation of average and maximal extension rates of RT synthesis under the alternative divalent cations

**Figure 1:**
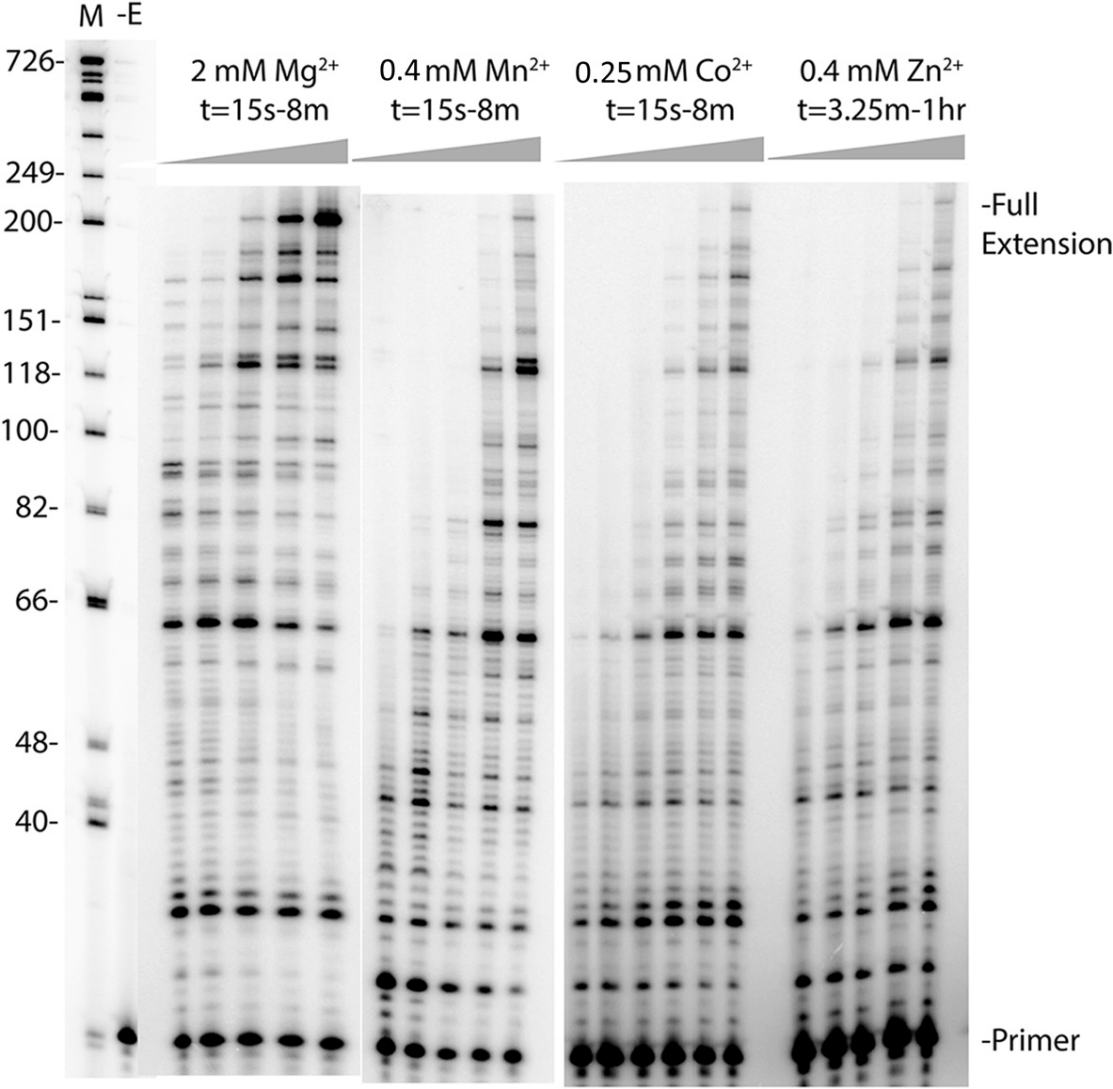
Time course of HIV RT synthesis on the ~760 nt RNA template used in the PCR-based α-complementation assay. Shown is an autoradiogram with extension of a 20 nt 5′ P-32 end-labeled DNA primer on the RNA template used for round 1 synthesis by HIV RT (see Figure 2). Full extension of the primer resulted in a 199 nt product. A DNA ladder with nt size positions is shown on the left. Concentrations of the cations and dNTPs are indicated above the lanes. Reactions were performed for (1-r) 15 s, 30 s, 1 min, 2 min, 4 min, or 8 min with Mg^2+^, Mn^2+^, and Co^2+^, and 3.25 min, 7.5 min, 15 min, 30 min, and 1 hour with Zn^2+^. A minus enzyme control (−E) is also shown. Refer to Materials and Methods for details.

Optimal extension conditions for HIV RT with Mg^2+^, Mn^2+^, Co^2+^, and Zn^2+^ in presence of 100 μM dNTPs were determined on a 425 nt RNA template derived from the *gag-pol* region of the HIV genome (as described in [20]). Optimal extension for each cation in the presence of 100 μM of each dNTP was observed at the following concentrations: 2 mM Mg^2+^, 0.4 mM Zn^2+^, 0.4 mM Mn^2+^, and 0.25 mM Co^2+^. Since a total concentration of 400 μM total nts (100 μM each) was used in the assays, the free concentration of each cation for optimal extension was ~1.6 mM for Mg^2+^, 0.15 mM for Zn^2+^, 0.15 mM for Mn^2+^, and 0.07 mM for Co^2+^. Note that all 3 alternative cations showed maximal activity at much lower concentrations than Mg^2+^. This suggests that these alternative cations bind more tightly to RT than the physiological cation. Interestingly, we also found that Cu^2+^ supported RT catalysis but optimum extension occurred at a much higher concentration of 3 mM (data not shown). Average and maximum extension rates were then calculated as described in Materials and Methods using the RNA template used for round 1 synthesis of the PCR-based *lacZα*-complementation fidelity assay. As expected, the rate of synthesis was fastest using Mg^2+^ and slowest with Zn^2+^ (Figure 1 and Table 1). An average extension rate of 1.8 ± 0.48 nts/s and a maximal extension rate of 7.4 ± 1.9 nts/s was observed with 2 mM Mg^2+^, whereas with 0.4 mM Zn^2+^, extension rates were 0.03 ± 0.02 nts/s and 0.19 ± 0.10 nts/s, respectively. Both 0.4 mM Mn^2+^ and 0.25 mM Co^2+^ decreased the average and maximal rate of extension as well (Table 1).

**Table 1:** **Synthesis rate on the RNA template for the PCR-based α-complementation fidelity assay at different cation concentrations**

**Cation**	^**a**^ **Optimal concentration**	^**b**^ **Maximal extension rate**	^**b**^ **Average extension rate**
	**mM**	**nts/sec**	**nts/sec**
Mg^2+^	2 (1.6)	7.4 ± 1.9	1.8 ± 0.5
Mn^2+^	0.4 (0.15)	1.2 ± 0.3	0.48 ± 0.09
Co^2+^	0.25 (0.07)	1.1 ± 0.1	0.23 ± 0.05
Zn^2+^	0.4 (0.15)	0.19 ± 0.10	0.03 ± 0.02

^a^The concentration of free cation is shown in parenthesis. Free cation concentration for Mg^2+^ was calculated as described in Materials and Methods using the dissociation constant for Mg^2+^ and ATP. Free concentrations for alternative cations (Mn^2+^, Co^2+^, and Zn^2+^) were approximately using the dissociation constant for Mg^2+^ and ATP.

^b^Values are averages from 3 experiments ± standard deviation. Rates were calculated as described in Materials and Methods.

### HIV RT shows greater fidelity with Zn^2+^ in the PCR-based and plasmid-based *lacZα*-complementation fidelity assays

**Figure 2:**
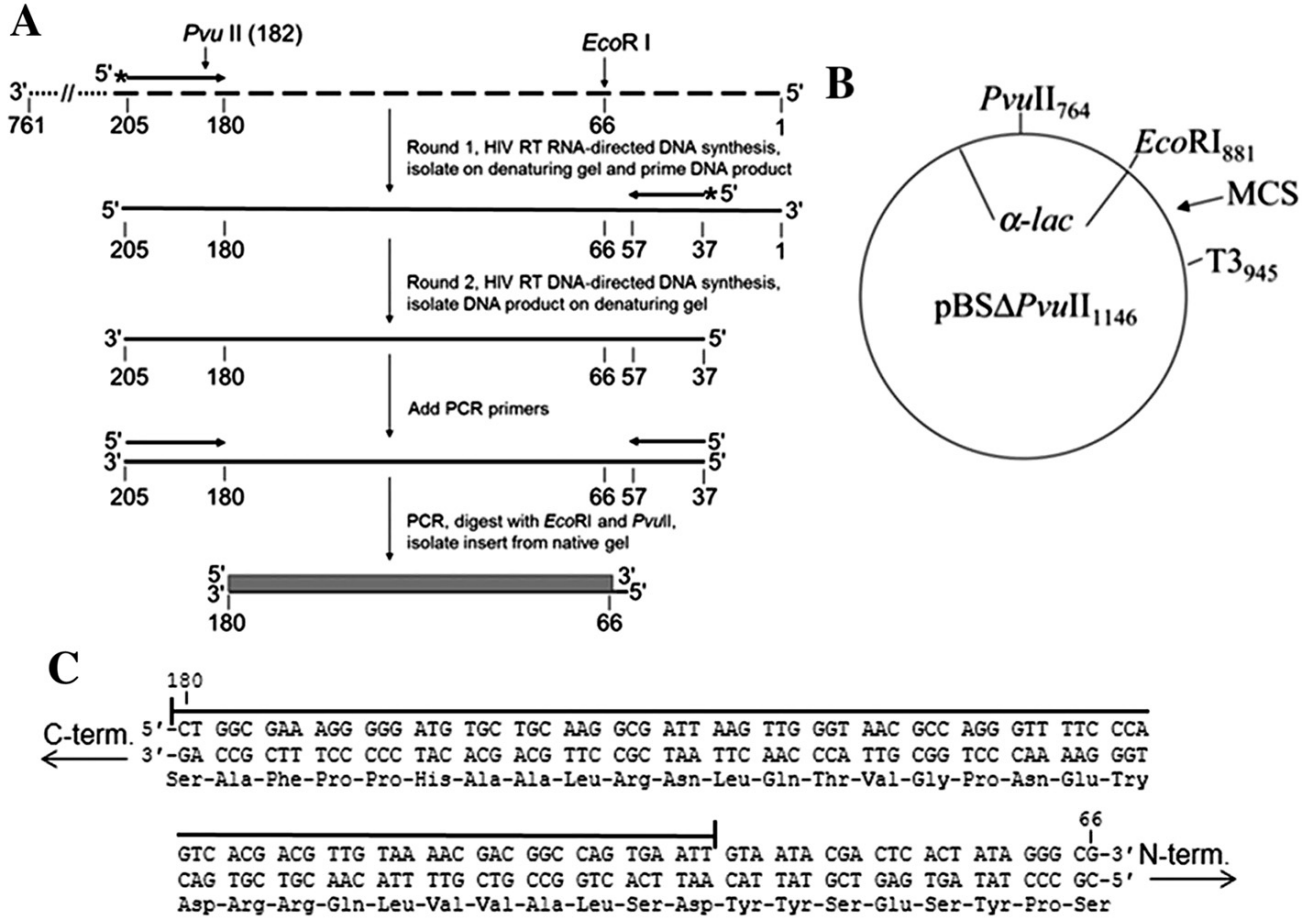
PCR-based *lacZα*-complementation system used to determine the fidelity of HIV RT. **(A)** An overview of the procedure used to assess polymerase fidelity is presented. RNA is represented by broken lines and DNA is represented by solid line. Primers have arrowheads at the 3′ end. The ~760 nt template RNA used as the initial template for HIV RT RNA-directed DNA synthesis is shown at the top with the 3′ and 5′ ends indicated. The positions of PvuII and EcoRI restriction sites are indicated for reference to the vector. The filled box at the bottom of the figure is the 115 base region of the *lacZ*α gene that was scored in the assay. Details for specific steps are provided under Materials and Methods. **(B)** Plasmid pBSM13ΔPvuII_1146_, is shown. Relevant sites on the plasmid are indicated and numbering is based on the parent plasmid (pBSM13+ (Stratagene)). **(C)** The nt and amino acid sequence for the 115 base region of the *lacZ*α gene that was scored in the assay is shown. Both strands of the DNA plasmid are shown since HIV RT synthesis was performed in both directions (see Figure 2A). A line is drawn above the 92 nts that are in the detectable area for substitution mutations while frameshifts can be detected over the entire 115 nt region. Based on a previous cataloging of mutations in this gene [51], the assay can detect 116 different substitutions (33.6% of the 345 possible substitutions in the 115 nt sequence) and 100% of the frameshift mutations.

The PCR-based assay was a modified version of an assay used previously to examine the fidelity of poliovirus 3Dpol [49,50] (Figure 2). The 115 nt region screened for mutations is shown in Figure 2C. The assay is capable of detecting all frameshift mutations and several substitutions (see legend) in this region [51]. The assay essentially mimics the reverse transcription process since both RNA- and DNA-directed RT synthesis steps are performed. Most of the possible background mutations can be accounted for by performing a control in which plasmid DNA is PCR amplified to produce an insert identical to those produced in the complete assay. These inserts should comprise all error sources except the errors derived from HIV RT and T3 RNA polymerase. An average background colony mutant frequency (CMF, number of white or faint blue colonies divided by total colonies) of 0.0019 ± 0.0014 was obtained (Table 2). This corresponds to 1 white or faint blue colony in every ~500 colonies. Further details of this assay are discussed in a recent publication by our group [48].

**Table 2:** **Colony mutation frequencies in PCR-based** ***lacZα*** **-complementation assay**

^**a**^ **Exp#**	^**b**^ **Bkg (CMF × 10** ^**−3**^ **)**	**2 mM MgCl**_**2**_	**0.4 mM ZnCl**_**2**_	**0.25 mM CoCl**_**2**_
		^**c**^ **1.6 mM free**	**0.15 mM free**	**0.07 mM free**
		**(CMF × 10** ^**−3**^ **)**	**(CMF × 10** ^**−3**^ **)**	**(CMF × 10** ^**−3**^ **)**
^d^1	4/1205	10/960	7/1328	
	3.3	10 (6.7)	5.3 (2.0)	
2	3/1726	13/1826	5/1315	16/1639
	1.7	7.1 (5.4)	3.8 (2.1)	9.8 (8.1)
3	1/1577	13/1899	6/2195	11/1520
	0.6	6.8 (6.2)	2.7 (2.1)	7.2 (6.6)
4	7/2942	26/3318	18/2977	
	2.4	7.8 (5.4)	6.0 (3.6)	
^e^Avg. ± S.D.	1.9 ± 1.4	7.9 ± 1.4	4.5 ± 1.5	8.5 ± 1.8
		(5.9 ± 0.6)	(2.5 ± 0.8)	(7.4 ± 1.1)
^f^P-value			4.4 x 10^−4^	0.098
^g^Relative fidelity		1.0	2.4	0.8
^h^Tukey HSD		P < 0.01 (Zn^2+^)	P < 0.01 (Mg^2+^)	P < 0.01 (Zn^2+^)
		P- N.S. (Co^2+^)	P < 0.01 (Co^2+^)	P- N.S. (Mg^2+^)

^a^Independent experiments performed at different times. In typical experiments, 1000–3500 colonies were scored for each condition.

^b^In background assays, plasmid pBSM13ΔPVUII (Figure, 1B) was used as a template in PCR reactions to generate the insert that was scored in the assays. Numbers shown are the “colony mutation frequency” (CMF) defined as white + faint blue colonies divided by total colonies. Refer to the Results and Methods sections for details.

^c^Free cation concentration under each condition was calculated as described in Materials and Methods using the dissociation constant for Mg^2+^ and ATP.

^d^Numbers shown on top are: (white + faint blue colonies)/total colonies. The bottom number is the colony mutation frequency (CMF) (see b above) for experiments under the listed condition. The CMF minus the background frequency from column two is in parentheses.

^e^Averages ± standard deviations from the experiments in the column are shown.

^f^Values were calculated using a standard Student’s t-test and the background subtracted values from each condition. All values were compared to the 2 mM Mg^2+^ condition.

^g^All values are relative to the 2 mM MgCl_2_ average CMF-Bkg. value (0.0059). The 0.0059 value was divided by the average CMF-Bkg. value for each condition to determine relative fidelity. Higher numbers indicate greater fidelity.

^h^In order to address the effect of comparing multiple sample conditions on statistical significance, ANOVA analysis coupled with a Tukey’s honest significance of difference (Tukey HSD) test were conducted using the background subtracted values from all samples and the calculator available online from Statistica: http://statistica.mooo.com/OneWay_Anova_with_TukeyHSD. The cation in parenthesis is being compared to the one listed in the column. N.S.- Not Significant.

Using 2 mM Mg^2+^, a CMF value of 0.006 (about 1 mutant colony in every 167 total) was obtained after background subtraction (Table 2). Results using Co^2+^ were similar to Mg^2+^ while Zn^2+^ increased fidelity about 2.5-fold (with high statistical significance). Although Co^2+^ is reported to be mutagenic, its effect on the mutation rate of polymerases is concentration-dependent [40,42,46]. For example, the error frequency of avian myeloblastosis virus (AMV) RT increased from about 1 error per 1680 nt additions with Mg^2+^ to 1 error per 1100 nt addition with activating concentrations of Co^2+^ (1 mM), but increased further to 1 error per 200 nt addition when excess amounts of Co^2+^ were used (5 mM) [40]. Only 0.07 mM free Co^2+^ was used in these assays and it is possible that Co^2+^ does not have a profound impact on fidelity at this concentration. This was further tested in the gapped plasmid assay described below.

A second gapped plasmid-based *lacZα-*complementation fidelity assay, similar to the phage-based *lacZα* gap-filling assay, was performed to further confirm results obtained from the PCR-based assay. The gap filled by the polymerase is in a plasmid construct, which after fill in, is directly transfected into bacteria. Bacterial colonies rather than phage plaques are scored by blue-white screening in this assay. This assay screens a large region (288 nts) of the *lacZα* gene including the promoter sequence and it avoids the enzymatic (T3 RNA polymerase and *Pfu* polymerase) background issues of the PCR-based assay. The results (Table 3) were in strong agreement with the PCR-based assay (Table 2). In this assay, Mg^2+^ was modestly more accurate than 0.25 mM Co^2+^, while Zn^2+^ once again showed ~2.5–fold greater fidelity than Mg^2+^. Interestingly, Mn^2+^, a known pro-mutagenic cation for several polymerases including HIV RT [43], was comparable to Mg^2+^ in the assays when used at its optimal concentration (0.4 mM total and 0.15 mM free). However, both Co^2+^and Mn^2+^ were highly mutagenic when used at 6 mM, an amount which is in the same range shown by others to decrease the fidelity of several polymerases *in vitro* [39-43,52]. There was a ~25-fold decrease in fidelity with 6 mM Mn^2+^ compared to 0.4 mM Mn^2+^. Similarly, a ~7-fold decrease in fidelity was observed with 6 mM vs. 0.25 mM Co^2+^. Both cations also showed severely inhibited polymerase activity at the 6 mM concentration while Zn^2+^ incorporates only a few nts even after prolonged incubation at high concentrations (see [4], results with Co^2+^ were similar to those shown with Mn^2+^ in this report). Overall, the results from both the PCR-based and gapped plasmid-based *lacZα-*complementation fidelity assays show that the fidelity of RT increases with Zn^2+^ and presumed pro-mutagenic cations do not modify RT’s error rate significantly when used at low concentrations optimal for catalysis.

**Table 3:** **Colony mutant frequencies in plasmid –based** ***lacZα*** **-complementation assay**

^**a**^ **Exp#**	^**b**^ **Bkg (CMF × 10** ^**−3**^ **)**	**2 mM MgCl**_**2**_	**0.4 mM MnCl**_**2**_	**6 mM MnCl**_**2**_	**0.25 mM CoCl**_**2**_	**6 mM CoCl**_**2**_	**0.4 mM ZnCl**_**2**_
		^**c**^ **1.6 mM free**	**0.15 mM free**	**5.6 mM free**	**0.07 mM free**	**5.6 mM free**	**0.15 mM free**
		**(CMF × 10** ^**−3**^ **)**	**(CMF × 10** ^**−3**^ **)**	**(CMF × 10** ^**−3**^ **)**	**(CMF × 10** ^**−3**^ **)**	**(CMF × 10** ^**−3**^ **)**	**(CMF × 10** ^**−3**^ **)**
^d^1	4/1893	11/1395	10/1297				10/2993
	2.1	7.9 (5.8)	7.7 (5.6)				3.3 (1.2)
2	2/1246	8/1176	7/1067		10/1054		5/1301
	1.6	6.8 (5.2)	6.6 (5.0)		9.5 (7.9)		3.8 (2.2)
3	3/3456	10/1755	10/2145		16/1389		14/3799
	0.9	5.7 (4.8)	4.7 (3.8)		12 (11.1)		3.7 (2.8)
4	4/1250	10/1192	11/1257		12/1007		
	3.2	8.4 (5.2)	8.8 (5.6)		12 (8.8)		
5	3/3113			160/1823	14/1324	101/1084	
	0.96			88 (87)	11 (10)	93 (92)	
6	7/5866			249/1286		90/1850	
	1.2			194 (193)		49 (48)	
7	3/2682			375/2740		119/2503	
	1.1			137 (136)		48 (47)	
^e^Avg. ± S.D.	1.6 ± 0.8	7.2 ± 1.7	7.4 ± 1.8	140 ± 53	11.1 ± 1.2	63 ± 26	3.6 ± 0.3
		(5.3 ± 0.4)	(5.7 ± 1.6)	(139 ± 53)	(9.5 ± 1.4)	(62 ± 26)	(2.1 ± 0.8)
^f^Statistics (see legend)			0.63	0.0034	0.0012	0.0059	0.00097
^g^Relative fidelity		1	0.93	0.038	0.56	0.085	2.5

^a^Independent experiments performed at different times.

^b^In background assays, the gapped plasmid was transformed into the bacteria allowing the bacterial polymerases to fill in the gap. Numbers shown are the “colony mutant frequency” (CMF) defined as white + faint blue colonies divided by total colonies. Refer to the Results and Methods sections for details.

^c^Free cation concentration under each condition was calculated as described in Materials and Methods using the dissociation constant for Mg^2+^ and ATP.

^d^Numbers shown on top are: (white + faint blue colonies)/total colonies. The bottom number is the colony mutant frequency (CMF) (see b above) for experiments under the listed condition. The CMF minus the background frequency from column two is in parentheses.

^e^Averages ± standard deviations from the experiments in the column are shown. Values in parentheses are after background subtraction.

^f^P-values shown were calculated using a standard Student’s t-test and the background subtracted values from each condition. All values were compared to the 2 mM MgCl_2_ condition. In order to address the effect of comparing multiple sample conditions on statistical significance, ANOVA analysis coupled with a Tukey’s honest significance of difference (Tukey HSD) test were conducted using the background subtracted values from samples and the calculator available online from Statistica:http://statistica.mooo.com/OneWay_Anova_with_TukeyHSD. The 6 mM MnCl_2_ and CoCl_2_ conditions were excluded from the analysis as their dramatically different magnitudes compared to other values complicates the analysis. Tukey analysis indicated highly significant differences (P < 0.01) for all conditions tested except 2 mM MgCl_2_ vs. 0.4 mM MnCl_2_, which was not significant, consistent with the insignificant P value (0.63) from the Student’s t-test.

^g^All values are relative to the 2 mM Mg^2+^ average CMF-Bkg. value (0.0053). The 0.0053 value was divided by the average CMF-Bkg. value for each condition to determine relative fidelity. Higher numbers indicate greater fidelity.

### Estimation of mutation frequency from CMF and sequencing data

**Figure 3:**
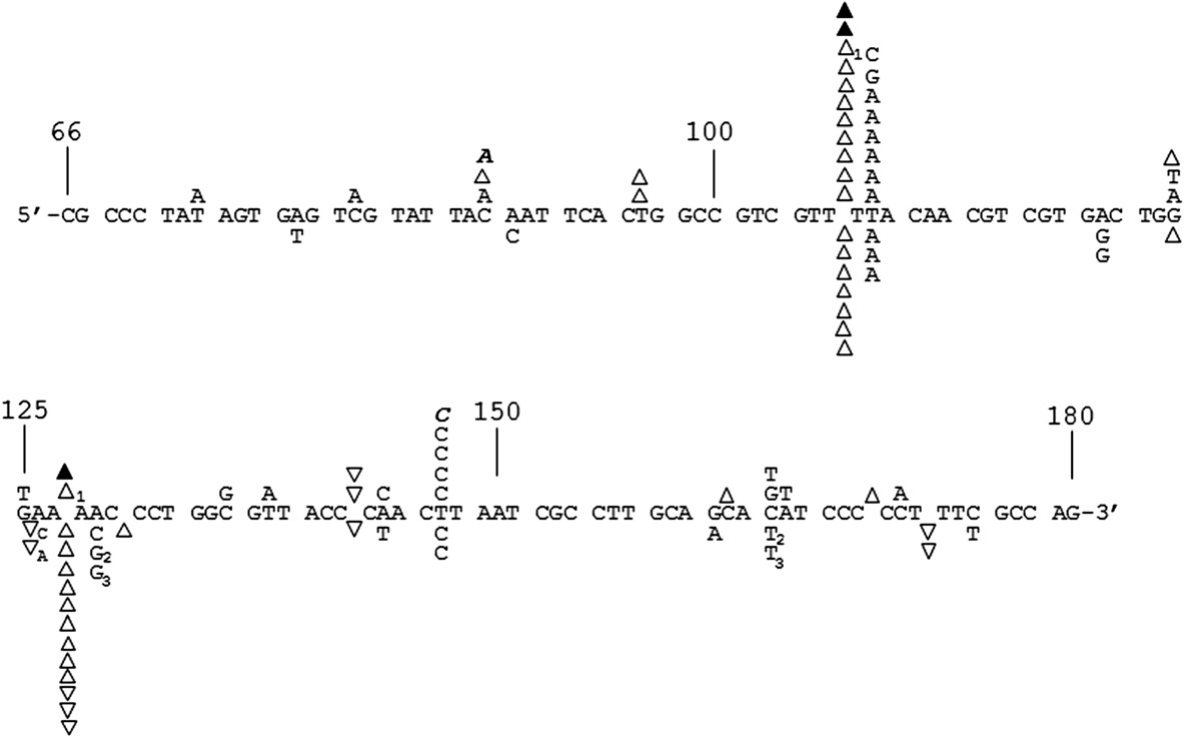
DNA sequence analysis from the PCR-based *lacZα*-complementation fidelity assay. The 115 base region analyzed for mutations is shown. The coding strand for *lacZα* is shown in the 5-3′ direction (bottom strand in Figure 2C). Numbering is as shown in Figure 2C. Deletions are shown as regular triangles, insertions are shown as downward triangles with the inserted base shown adjacent to the downward triangle, unless it was the same as the base in a nt run, and base substitutions are shown directly above or below the sequence. Substitutions shown correspond to the recovered sequence for the coding strand; however, these mutations could have occurred during synthesis of the non-coding strand as well (i.e. a C to A change shown here could have resulted from a C to A change during synthesis of the coding strand or a G to T during synthesis of the non-coding strand) (see Figure 2). Mutations recovered from HIV RT with 2 mM Mg^2±^, and mutations from background controls are shown above the sequence as open triangles and normal text or filled triangles and bold italicized text, respectively. Mutations from HIV RT at 0.4 mM Zn^2+^ are shown below the sequence. Individual sequence clones which had multiple mutations (more than one mutation event) are marked with subscripts adjacent to the mutations. Several clones with deletions (either single or multiple deletions) at positions 181–183, just outside of the scored region were also recovered (not shown). This was the dominant mutation type recovered in background controls (19 out of 24 total sequences) and probably resulted from improper ligation events or damaged plasmid vectors (see [48]).

An estimate of the base misincorporation frequency can be made from the CMFs in Table 2 and the sequencing results in Figure 3 as described before [48]. In experiments with Mg^2+^, ~41% (17/42) of recovered mutations, after excluding the background mutations, were insertions or deletions (indels), and ~59% (25/42) substitutions. Using a 33.6% detection rate for substitutions and 100% detection rate for indels in this region (see Figure 2C and accompanying legend) and a CMF of 0.0059 (from Table 1), the mutation frequency for Mg^2+^ was 5.6 × 10^−5^, or ~1 error per 18,000 incorporations ((0.0059 × 0.41)/230 = 1.1 × 10^−5^ for indels, and ((0.0059 × 0.59)/230)/0.336 = 4.5 × 10^−5^ for substitutions, total is 5.6 × 10^−5^ for both (see Figure 3 legend for further details)). Synthesis with Zn^2+^ resulted in a higher ratio of indels vs. substitution: indels ~63% (26/41), and ~37% substitutions (15/41) were obtained. With a CMF of 0.0025 (Table 1), a mutation frequency of 1.9 × 10^−5^ or ~ 1 error per 53,000 incorporations was obtained for experiments with Zn^2+^. This value is also closer to the rate of ~1 error per 77,000 incorporations that was observed with more physiological (0.25 mM), though sub-optimal Mg^2+^ concentrations [4].

It is also possible to estimate the mutation frequency using the plasmid-based assay results (Table 3). As no sequencing data was acquired, a combined error rate for both substitutions and indels can be estimated using the formula: $ER = \frac{CMF}{D \times P}$, where ER is the error rate, CMF is the Colony Mutant Frequency (from Table 3), D is the total number of detectable sites for plasmid pSJ2 which is 448, P is the expression frequency of the plasmid which equals 0.444 [53]. The calculations yield a mutation rate of 2.7 × 10^−5^ for 2 mM Mg^2+^ and 1.1 × 10^−5^ for Zn^2+^. A mutation rate of 2.9 × 10^−5^ for 0.4 mM Mn^2+^ vs. 7.0 × 10^−4^ for 6 mM Mn^2+^ (~24-fold increase) and 4.8 ×10^−5^ for 0.25 mM Co^2+^ vs. 3.1 × 10^−4^ for 6 mM Co^2+^ (~7-fold increase) was obtained. Both the PCR-based and plasmid-based assays showed a comparable fidelity increase (~2.5-fold) for Zn^2+^ vs. Mg^2+^, although the calculated mutation frequency rates were modestly lower in the plasmid-based assay.

### Analysis of fidelity by steady-state kinetics also demonstrates higher fidelity with Zn^2+^

**Figure 4:**
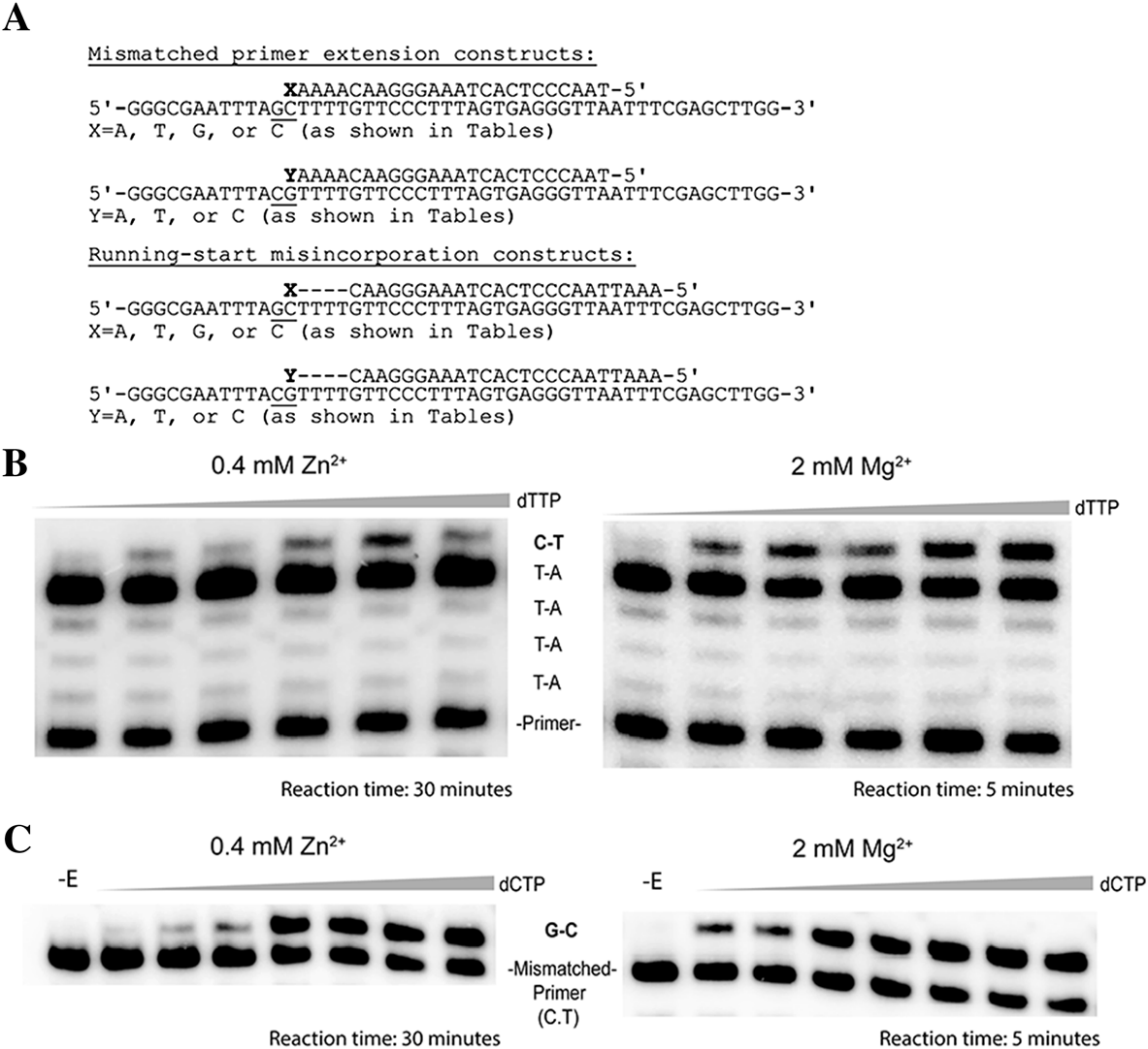
A, B and C. Sequences used in mismatched primer extension and running-start misincorporation assays and examples of analysis. **(A)** The sequence of the DNA used in each assay type is shown. The underlined nts show the only differences between the two templates. Only one primer was used in the running-start assays and it terminated at the 3′ C nt before the dashes. The four dashes indicate the 4 A nts that must be incorporated before RT incorporates the target nt (denoted by X or Y). **(B)** Running-start misincorporation of C.T base pair at 2 mM Mg^2+^ or 0.4 mM Zn^2+^. Reactions were performed on the primer-template shown in panel A for the indicated time with a final free concentration of 2 mM Mg^2+^ or 0.4 mM Zn^2+^ (adjusted according to the total concentration of dNTPs in each reaction using the *K*_*d*_ value of Mg^2+^ and ATP). A fixed concentration of dATP = 55 μM was used in all running-start reactions for elongation of the primer to the target site. The concentration of the target nt (dTTP for C.T insertion) in each lane was from l-r: 400, 630, 1380, 2610, and 3660 μM. For other base pair misinsertions noted in the Table 4, the target nt was changed according to the desired misinsertion. **(C)** Extension of a mismatched primer-template with a C.T 3 terminus, using 2 mM Mg^2+^ and 0.4 mM Zn^2+^. Reactions were performed on the primer-template shown in panel A for the indicated time with the same free cation concentration as above. The concentration of the next correct nt (dCTP) in each lane was from l-r: 50, 100, 200, 400, 630, 1200 and 1870 μM. -E lane corresponds to no enzyme added.

Kinetic assays have been used by many groups as a reliable way to estimate polymerase fidelity by insertion of specific nt mismatches or extension of specific mismatched primer termini (reviewed in [54-56]). Although pre-steady-state assays are more useful for understanding kinetic parameters for misincorporation, steady-state assays are much simpler to perform and typically yield results that are broadly similar to results with pre-steady-state assays [54]. Mismatched primer extension and running-start assays using the sequences shown in Figure 4 were performed with constant concentrations of free cation of 0.4 mM Zn^2+^ or 2 mM Mg^2+^. Note that reactions with each cation were performed using different enzyme concentrations and time points (see Materials and Methods). This was necessary as catalysis with Zn^2+^ is much slower than Mg^2+^ yielding negligible levels of extension with the conditions used for Mg^2+^. The results with Zn^2+^ were seemingly consistent with steady-state conditions as the reactions were conducted over a prolonged period (30 min) and the ratio of unextended to extended primers remained high (i.e. the substrate was not significantly depleted). However, it is possible that these reactions may to some extent reflect pre-steady-state conditions, to an extent, since RT-primer-template complexes are extremely stable in Zn^2+^ while catalysis is slow [20]. Because of these constraints, a direct comparison of the kinetic and equilibrium constants between the two cations cannot be made. However, the fidelity with Zn^2+^ relative to that with Mg^2+^ can still be estimated by comparing misinsertion ratios calculated for particular mismatches with each cation. The running-start assays performed here test RT’s ability to misincorporate at a template C or G residue (depending on the sequence) after a “running-start” on a run of T’s immediately downstream of the primer 3′ terminus. Experiments were analyzed on denaturing polyacrylamide-urea gels (Figure 4B). A statistically pronounced (based on P-values) increase in fidelity was observed for all mismatches with Zn^2+^ (Table 4). The misinsertion ratio for the G.A mismatch, which is a difficult mismatch to make [48], could not be evaluated with Zn^2+^ as no incorporation was detected (data not shown). In general, there was a ~4-fold to 8-fold increase in fidelity for different mismatches in Zn^2+^ compared to Mg^2+^.

**Table 4:** **Running-start misincorporation assay of various mismatches with Mg** ^**2+**^ **or Zn** ^**2+**^

^**a**^ **Cation**	^**b**^ **Base pair**	^**c**^ ***V***_***max***,***rel***_	^**d**^ ***K***_***m***_	***V***_***max***_/***K***_***m***_	^**e**^ **Misinsertion ratio,** ***f***_***ins***_	^**f**^ **Relative fidelity**	^**g**^ **P-value**
			**μM**	**μM** ^**−1**^			
^h^Mg^2+^	C.G	2.9 ± 1.1	0.68 ± 0.33	4.3 ± 1.3	1		
	G.C	1.4 ± 0.26	1.2 ± 0.64	1.2 ± 0.6	1		
	C.T	0.55 ± 0.14	1863 ± 670	2.9 (±0.33) x 10^−4^	6.7 (±2.6) x 10^−5^		
	C.A	0.50 ± 0.06	825 ± 217	6.1 (±1.1) x 10^−4^	1.4 (±0.3) x 10^−4^		
	C.C	0.09 ± 0.07	312 ± 166	2.9 (±0.8) x 10^−4^	6.7 (±1.9) x 10^−5^		
	G.T	0.36 ± 0.16	242 ± 50	1.5 (±0.9) x10^−3^	1.3 (±0.8) x10^−3^		
	G.A	0.38 ± 0.11	1515 ± 85	2.5 (±0.6) x 10^−4^	2.1 (±0.5) x 10^−4^		
Zn^2+^	C.G	8.2 ± 5.5	0.19 ± 0.09	43 ± 11	1		
	G.C	5.8 ± 5.1	5.1 ± 4.7	1.3 ± 0.6	1		
	C.T	0.19 ± 0.07	244 ± 102	7.8 (±0.97) x 10^−4^	1.8 (±0.8) x 10^−5^	3.7	0.028
	C.A	0.55 ± 0.33	518 ± 43	1.1 (±0.6) x 10^−3^	2.5 (±1.6) x 10^−5^	5.6	0.0023
	C.C	0.05 ± 0.01	224 ± 104	2.2 (±1.4) x 10^−4^	5.1 (±5.6) x 10^−6^	13.1	0.029
	G.T	0.2 ± 0.08	914 ± 378	2.2 (±0.7) x 10^−4^	1.6 (±0.6) x 10^−4^	8.1	0.038
	G.A	N.D.					

^a^The extension reactions were carried as described in Materials and Methods using either 2 mM free Mg^2+^ or 0.4 mM free Zn^2+^. Free cation concentration was calculated as described in Materials and Methods using the dissociation constant for Mg^2+^ and ATP. All values are averages from at least 3 experiments ± standard deviation.

^b^Refer to the running-start sequences in Figure 2. The particular mismatch that was measured after incorporation of a run of A’s over a run of T’s on the template is shown in the column

^c^ *V*_*max*,*rel*_ = *I*_*i*_/*I*_*i* − 1_ where *I*_*i*_ is the sum of band intensities at the target site and beyond, *I*_*i* − 1_ is the intensity of the band prior to the target band. See Materials and Methods for a description.

^d^Refers to the K_m_ of the nucleotide being incorporated at the target site (e.g. dGTP for C.G and dATP for C.A).

^e^ *f*_*ins*_ is the ratio of {*V*_*max*_/*K*_*m*_ (*mismatch*)}/{*V*_*max*_/*K*_*m*_ (*match*)}.

^f^Fidelity values for misincorporation in Zn^2+^ are relative to the same mismatch using Mg^2+^. Determinations were made by dividing the misinsertion ratio in Mg^2+^ by the ratio in Zn^2+^. Higher values indicate greater fidelity.

^g^Values were calculated using a standard Student’s t-test. Misinsertion ratio values from experiments in Zn^2+^ were compared against the Mg^2+^ condition for the same misincorporation.

^h^Values for Mg^2+^ were taken from (33).

The ability of RT to extend primers with mismatched 3′ termini in Mg^2+^ or Zn^2+^ was also evaluated (Figure 4C). Extension was more difficult with Zn^2+^ and the magnitude of the difference was dependent on the particular mismatch (Table 5). Fidelity increased between ~3- fold to 5- fold for C.T, C.A, and C.C mismatches, however the G.T mismatch showed a statistically insignificant (P-value of 0.375) change in fidelity. Consistent with results in the running-start reaction, no extension of the G.A mismatched primer was detected with Zn^2+^. Overall, results from running-start and mismatch extension assays are in strong agreement with the *lacZα*-complementation assays showing that fidelity with HIV RT improves in Zn^2+^.

**Table 5:** **Mismatched primer extension with Mg** ^**2+**^ **or Zn** ^**2+**^

^**a**^ **Cation**	^**b**^ **Base pair at the 3′ end**	^**c**^ ***V***_***max***_	^**d**^ ***K***_***m***_	***V***_***max***_ **/** ***K***_***m***_	^**e**^ **Standard extension efficiency,** ***f***_***ext***_	^**f**^ **Relative fidelity**	^**g**^ **P-value**
		**%/min**	**μM**				
^h^Mg^2+^	C.G	21.4 ± 2.0	0.37 ± 0.13	57.8 ± 19.2	1		
	G.C	39.9 ± 14.5	0.27 ± 0.13	147.8 ± 40.6	1		
	C.T	22.5 ± 2.1	396 ± 28	5.7 (±0.9) x 10^−2^	9.9 (±1.7) x 10^−4^		
	C.A	11.1 ± 2.9	445 ± 188	2.5 (±0. 57) x 10^−2^	4.3 (±0.94) x 10^−4^		
	C.C	0.70 ± 0.29	77 ± 14	9.1 (±2.2) x 10^−3^	1.6 (±0.38) x 10^−4^		
	G.T	18.6 ± 3.3	157 ± 44	0.12 ± 0.03	8.1 (±2.5) x10^−4^		
	G.A	0.95 ± 0.04	196 ± 50	4.8 (±1.6) x 10^−3^	3.2 (±1.0) x 10^−5^		
Zn^2+^	C.G	0.62 ± 0.23	0.04 ± 0.01	15.5 ± 5.8	1	1	
	G.C	0.39 ± 0.10	0.04 ± 0.01	9.8 ± 1.9	1	1	
	C.T	2.9 ± 1.0	591 ± 63	4.9 (±2. 3) x 10^−3^	3.2 (±1.6) x 10^−4^	3.1	0.026
	C.A	3.3 ± 1.0	1240 ± 186	2.7 (±1.3) x 10^−3^	1.7 (±0.75) x 10^−4^	2.5	0.029
	C.C	0.50 ± 0.14	914 ± 457	5.4 (±4.6) x 10^−4^	3.5 (±3.4) x 10^−5^	4.6	0.023
	G.T	6.8 ± 1.8	1223 ± 568	5.6 (±2.8) x 10^−3^	5.7 (±3.2) x 10^−4^	1.4	0.375
	G.A	N.D.					

^a^The extension reactions were carried out in either 2 mM fee Mg^2+^ or 0.4 mM free Zn^2+^. Free cation concentration was calculated as described in Materials and Methods using the dissociation constant for Mg^2+^ and ATP. All values are averages from at least 3 experiments ± standard deviation.

^b^Refer to the mismatch extension sequences in Figure 2. In this assay primers with a matched C.G or a mismatched C.T, C.A, or C.C at the 3′ end were extended on one seqeunce. A second sequence with a matched G.C or mismatched G.T or G.A was also used.

^c^ *V*_*max*_ is the maximum velocity of extending each primer- template hybrid. See Materials and Methods for a description.

^d^Refers to the *K*_*m*_ of the next correct nucleotide being added (i.e. dCTP for C.G, C.T, C.A, and C.C or dGTP for G.C, G.T, and G.A extensions).

^e^ *f*_*ext*_ is the ratio of {*V*_*max*_/*K*_*m*_ (*mismatch*)}/{*V*_*max*_/*K*_*m*_ (*match*)}.

^f^Fidelity values for misextension in Zn^2+^ are relative to the same mismatch using Mg^2+^. Determinations were made by dividing the standard extension efficiency in Mg^2+^ by the same parameter in Zn^2+^. Higher values indicate greater fidelity.

^g^Values were calculated using a standard Student’s t-test. Standard extension efficiency values from experiments in Zn^2+^ were compared against the Mg^2+^condition for the same mismatch.

^h^Values for Mg^2+^ were taken from (33).

## Discussion

The results presented in this paper show that using concentrations optimized for catalysis, Zn^2+^ increases the fidelity of HIV-RT approximately 2-fold to 3-fold when compared to the physiological cation Mg^2+^. Mn^2+^ and Co^2+^ decreased the fidelity of RT at high concentrations; but at optimal concentrations these effects were almost completely mitigated. For Zn^2+^, misincorporation (as determined by running-start assays) and mismatch extension (as determined with mismatched primer-templates) were both influenced, suggesting that both steps involved in fidelity could be affected by Zn^2+^.

There may be several possible mechanisms by which Zn^2+^ alters fidelity. The geometry supported by different cations in the active site of polymerases has been proposed to affect fidelity. Magnesium supports tetrahedral symmetry at the active site, whereas Mn^2+^ accommodates square planar, octahedral, and tetrahedral symmetries (reviewed in [57]). The ability of Mn^2+^ to accommodate more than one type of symmetry may increase the reaction rate of misaligned substrates and hence decrease the fidelity of polymerization. It is important to note that although results presented here indicate Mn^2+^ is not highly mutagenic at optimal concentrations, this may not be the case for other polymerases. Crystal structures of polymerases with Zn^2+^ in the active site are not available, but Zn^2+^ has been crystallized in a distorted tetrahedral symmetry in erythrocyte carbonic-anhydrase [58], as well as in a near tetrahedral geometry in Zn^2+^ superoxide dismutase [59]. It is possible that Zn^2+^ supports a different geometry than Mg^2+^ in the active site and promotes a configuration of the amino acid residues which may be better suited to discriminate against misaligned substrates.

Results showed that the fidelity of HIV RT with Mn^2+^ and Co^2+^ was concentration-dependent, as we observed previously for Mg^2+^ [48]. Although Mn^2+^ is generally considered to be pro-mutagenic [57], the error frequency for several DNA polymerases usually increased as the Mn^2+^ concentration increased [52]. In one report, *E. coli* DNA polymerase I and mammalian DNA polymerase β both showed relatively high fidelity when lower concentrations (below ~100 μM) of Mn^2+^ were used, whereas higher concentrations lead to greater mutagenesis. The high concentrations correlated with Mn^2+^ binding to the single stranded template and possibly to secondary binding sites on the polymerase, raising the possibility that these factors promote the lower fidelity observed at high Mn^2+^ concentrations [60]. In this regard, *E. coli* DNA polymerase I has been reported to have as many as 21 Mn^2+^ binding sites on a single molecule but just a single high affinity binding site [61]. The effect, if any, of binding at the secondary sites is unknown. Still, when compared to Mg^2+^, careful analysis with other polymerases has suggested that Mn^2+^ is promutagenic over a range of concentrations [62]. Differences between our results and these may stem from intrinsic differences in the enzymes or the different nucleic acid substrate used (many of the former experiments used homopolymers). Also, unlike RT, most DNA polymerases have intrinsic exonuclease activity. El-Deiry *et al.* [62] found that *E. coli* DNA polymerase I demonstrated a significant reduction in 3′ to 5′ exonuclease proofreading activity in the presence of Mn^2+^. This effect exacerbated the accelerated misincorporation with Mn^2+^ which was observed.

It is also possible that Zn^2+^ affects the rate of conformational change in the enzyme and this leads to an alteration in fidelity. Catalysis with Zn^2+^ is extremely slow (Table 1 and [20]) even though the complex between the enzyme and primer-template is over 100 times more stable than with Mg^2+^ [20]. This indicates that one or more of the steps in catalysis is slow. Conformational transition of the protein after binding the substrate has a significant contribution to the ability of RT to add the correct substrate [63]. Upon binding the substrate in Mg^2+^, the enzyme undergoes a conformational change to reach the transition state. A correctly matched nt then leads to tight binding and alignment of catalytic residues to promote catalysis, whereas a mismatched nt does not induce the tight binding state, thereby facilitating the rapid opening of the specificity domain and release of the misaligned substrate [63]. The conformational change in the specificity subdomain (fingers subdomain) of the polymerase plays a key role in determining the enzyme fidelity, and it will be interesting to investigate if the modified catalysis with Zn^2+^ affects either the conformational change or the rate of conformational change in a way which might increase the specificity. Consistent with the model of slower catalysis promoting higher fidelity, suboptimal Mg^2+^ concentrations also enhanced fidelity [48]. The observed enhancement was similar to what was observed with Zn^2+^ as it resulted mostly due to a decrease in substitutions rather than insertion and deletion errors. Since insertions and deletions often result from primer-template slippage mechanisms, this suggests that both low Mg^2+^ and Zn^2+^ induce higher fidelity by intrinsically affecting RT catalysis rather than altering primer-template properties. It is possible that lowering the Zn^2+^ concentration to suboptimal levels could also alter fidelity, however, catalysis dramatically declines as the concentration of Zn^2+^ is either lowered or increased [20], making it difficult to test this possibility.

As was noted in the Introduction, the level of available Zn^2+^ and other divalent cations such as Mn^2+^ or Co^2+^ are kept extremely low in cells. Also, it is highly unlikely that these cations could support HIV replication. Although we show the alternative cations can support RT synthesis, the rate of nucleotide catalysis ranged from significantly reduced for Mn^2+^ and Co^2+^, to essentially negligible for Zn^2+^ (Table 1).

Finally, the possibly of using supplements or natural minerals including Zn^2+^ to treat HIV infection must be approached with caution. Low μM concentrations of Zn^2+^, which can inhibit HIV RT [20], are still ~2–3 orders of magnitude greater than the level of free available Zn^2+^ in cells. Low μM concentrations of free Zn^2+^ in cells could have profound effects on the transcription of specific genes and the oxidation state of cells. Nevertheless, Zn^2+^ as a constituent of cation based compounds like topical ointments for treating HIV infection still holds promise.

## Conclusions

In this report, we demonstrate that DNA synthesis by HIV RT in Zn^2+^ is slow, but highly accurate. It was even more accurate than with the physiologically relevant cation Mg^2+^, when both were used at optimal concentrations. Other presumably pro-mutagenic cations (Mn^2+^ and Co^2+^) showed fidelity levels that were comparable to Mg^2+^ under optimal conditions, while they were highly mutagenic when used at very high concentrations. This suggests that catalysis with these alternative cations is not intrinsically mutagenic and the observed mutagenicity in previous reports, may result from other mechanisms that could occur at high concentrations (see Discussion) that warrants further investigation.

## Methods

### Materials

Calf intestinal alkaline phosphatase (CIP), T3 RNA polymerase, “High Fidelity” (PvuII and EcoRI) and other restriction enzymes, T4 polynt kinase (PNK), and MuLV RT were from New England Biolabs. DNase (deoxyribonuclease)-free RNase (ribonuclease), ribonucleotides, and deoxyribonucleotides were obtained from Roche. RNase free-DNase I was from United States Biochemical. Rapid DNA ligation kit, RNasin (RNase inhibitor), and the phiX174 HinfI digest DNA ladder was from Promega. Radiolabeled compounds were from PerkinElmer. *Pfu* DNA polymerase was from Stratagene. DNA oligonucleotides were from Integrated DNA Technologies. G-25 spin columns were from Harvard Apparatus. RNeasy RNA purification and the Plasmid DNA Miniprep kits were from Qiagen. X-gal was from Denville Scientific, Inc. IPTG and media were from Gibco, Life Technologies. All other chemicals were obtained from Fisher Scientific, VWR, or Sigma. HIV RT (from HXB2 strain) was prepared as described [64]. The HIV RT clone was a generous gift from Dr. Michael Parniak (University of Pittsburgh). This enzyme is a non-tagged heterodimer consisting of equal proportions of p66 and p51 subunits. Aliquots of HIV RT were stored frozen at −80°C and fresh aliquots were used for each experiment.

### Polyacrylamide gel electrophoresis

Denaturing polyacrylamide gels (6, 8, and 16% w/v), native polyacrylamide gels (15% w/v), and 0.7% agarose gels were prepared and run as described [65].

### Preparation of RNA for the PCR-based *lacZα*-complementation fidelity assay and RNA-DNA hybridization

Transcripts (~760 nts) were prepared with T3 RNA polymerase and hybrids were prepared at a 2:1 5′ ^32^P-labeled primer:template ratio as previously described [49].

### Primer extension reactions for the PCR-based *lacZα*-complementation fidelity assay

For RNA-directed DNA synthesis, the ~760 nt RNA template was hybridized to a radiolabeled 25 nt DNA primer (5′-GCGGGCCTCTTCGCTATTACGCCAG-3′). Full extension produced a 199 nt final product (see Figure 2A). The long template was used to make it easier to separate DNA synthesis products from the RNA template on a denaturing polyacrylamide-urea gel (see below). The primer-template complex was pre-incubated in 48 μl of buffer (see below) for 3 min at 37°C. The reaction was initiated by addition of 2 μl of 5 μM HIV RT in 50 mM Tris–HCl pH 8, 80 mM KCl, 1 mM DTT and 10% glycerol and incubation was continued for 30 min for Mg^2+^, 1 hour for Mn^2+^ and Co^2+^, and 3 hours for Zn^2+^. Different time points were used to assure that all the reactions were essentially complete with each cation. The final concentration of reaction components were 200 nM HIV RT, 25 nM template, 50 nM primer, 50 mM Tris–HCl, 80 mM KCl, 1 mM DTT, 0.4% glycerol and 0.4 units/μl RNasin along with different concentrations of salts. A final concentration of 100 μM dNTPs was used along with one of the following divalent cation: 2 mM MgCl_2,_ 0.25 mM CoCl_2_, 0.4 mM ZnCl_2_. The final pH of the reactions was 7.7. After incubations, 1 μl of DNase-free RNase was added and the sample was heated to 65°C for 5 min. Typically two reactions for each condition were combined and material was recovered by standard phenol:chloroform extraction and ethanol precipitation. Pellets were resuspended in 20 μl of 10 mM Tris–HCl (pH 7) and 2X loading buffer (90% formamide, 10 mM EDTA (pH 8), 0.25% each bromophenol blue and xylene cyanol) and products were analyzed by gel electrophoresis on 6% polyacrylamide-urea gels (19:1 acrylamide:bis-acrylamide). Fully extended 199 nt DNA was located using a phosphoimager (Fujifilm FLA5100), and recovered by the crush and soak method [65] in 500 μl of elution buffer containing 10 mM Tris–HCl (pH 7). After overnight elution, this material was passed through a 0.45 μm syringe filter and recovered by ethanol precipitation after addition of 10% volume 3 M sodium acetate (pH 7) and 50 μg of glycogen. After centrifugation, the pellets were vigorously washed with 500 μl of 70% ethanol to remove any traces of EDTA that may have carried over from the gel and potentially interfere with the second round of synthesis. The recovered DNA was hybridized to another 20 nt radiolabeled DNA primer (5′-AGGATCCCCGGGTACCGAGC-3′) with 10-fold greater specific activity than the primer used for round one, and a second round of DNA synthesis was performed as described above except the reaction volume was 25 μl. Conditions for the cation, dNTPs, and pH were identical in the RNA- and DNA-templated reactions. Reactions were terminated with an equal volume of 2X loading buffer and products were gel purified as described above but on an 8% gel. The gel was run far enough to efficiently separate the 199 nt templates from the 162 nt full extension product of round 2.

### Polymerase chain reaction (PCR) for the PCR-based *lacZα*-complementation fidelity assay

The round two DNA (50% of recovered material) produced above by reverse transcription was amplified by PCR using the following primers: 5′-GCGGGCCTCTTCGCTATTACGCCAG-3′ and 5′- AGGATCCCCGGGTACCGAGC -3′. Reactions were performed and processed as previously described except that restriction digestion was done with 30 units each of “High Fidelity” EcoRI and PvuII in 50 μl of NEB buffer 3 for 2 hours at 37°C [49].

### Preparation of vector for PCR-based *lacZα*-complementation fidelity assay

Thirty μg of the plasmid pBSΔPvuII_1146_ [66] was double-digested with 50 units each of “High Fidelity” EcoRI and PvuII in 100 μl using the supplied buffer and protocol. After 3 hours, DNA was recovered by phenol-chloroform extraction and ethanol precipitation then treated with 20 units of CIP for 2 hours at 37°C in 100 μl of the supplied NEB restriction digest buffer 3. Dephosphorylated vector was recovered by phenol-chloroform extraction followed by ethanol precipitation and quantified using absorbance at 260 nm. The quality of the vectors for the fidelity assay was assessed in two ways: (a) ligation (see below) of the vector preparation in the absence of insert; and (b) re-ligation of the vector preparation and PvuII-EcoRI cleaved fragment (recovered from agarose gels after cleavage of pBSΔPvuII_1146_ but before dephosphorylation as described above). Vectors from (a) that did not produce any white or faint blue colonies and very few blue colonies in the complementation assay (see below), and those producing colony mutant frequencies of less than ~0.003 (1 white or faint blue colony in ~333 total) in (b) were used in the fidelity assays.

### Ligation of PCR fragments into vectors and transformation for the PCR-based lacZα-complementation fidelity assay

The cleaved vector (50 ng, ~0.025 pmol) and insert fragments (0.05 pmol) were ligated at a 1:2 (vector: insert) molar ratio using a rapid DNA ligation kit. Ligation and transformation of *E. coli* GC5 bacteria were carried out as previously described [49]. White or faint blue colonies were scored as harboring mutations while blue colonies were non-mutated. Any colonies that were questionable with respect to either being faint blue or blue were picked and replated with an approximately equal amount of blue colony stock. Observing the faint blue colony in a background of blue colonies made it easy to determine if the colony was faint blue rather than blue.

### Gapped plasmid-based based *lacZα*-complementation fidelity assay

The gapped version of the plasmid pSJ2 was prepared as described [53]. One nM of the gapped plasmid was filled by 100 nM RT at 37°C in 20 μl of buffer containing 50 mM Tris–HCl, 80 mM KCl, 1 mM DTT, 2 μg of bovine serum albumin, 100 μM dNTPs, and varying concentrations of different cations. The reaction pH was 7.7. Reactions with 2 mM Mg^2+^, 0.25 mM Mn^2+^, 6 mM Mn^2+^, 0.4 mM Co^2+^, and 6 mM Co^2+^ were carried out for 30 min while reactions with 0.4 mM Zn^2+^ were performed overnight. Reactions were terminated by heating at 65°C for 15 min. After confirming complete extension by restriction digestion analysis (see [53]), ~1 μl of the remaining original mixture was transformed into *E. coli* GC5 cells. The colony mutant frequency (CMF) was determined using blue-white screening as described above.

### Running-start misincorporation assays

The approach used for these assays was based on previous results [67]. Reactions were performed as above using the same template but with a primer (5′- GAAATTAACCCTCACTAAAGGGAAC -3′) (Figure 4A) which does not have the last five nts at the 3′ end and has 5 additional bases at the 5′ end. Reactions with 2 mM Mg^2+^ and 0.4 mM Zn^2+^ were performed for 3 min and 30 min respectively at 37°C. The nt directed by the homopolymeric T run on the template running (dATP) was kept at a constant saturating concentration (55 μM) and the nt to be misinserted (for example, dTTP for measuring C-T misinsertion kinetics) was added at increasing concentrations in these reactions. The reaction pH was 7.7. Reactions were initiated by adding 2 μl of HIV RT (final concentration of 2 nM for Mg^2+^ reactions and 8 nM for Zn^2+^ reactions) and terminated by adding 2X loading buffer. The reactions were then electrophoresed on 16% denaturing polyacrylamide gels, dried, and imaged using a Fujifilm FLA5100 phosphoimager. Steady-state kinetic parameters *K*_*m*_, and *V*_*max*_ were then calculated as described below. The amount of free cation in each reaction was adjusted according to the dNTP concentration because dNTPs are the major chelators of Mg^2+^ or Zn^2+^ in the reactions. The concentration of free cation was calculated using the formula:

$$ \left[ED\right]=0.5\left({E}_t + {D}_t + {K}_d\right)-0.5\left({\left({E}_t + {D}_t + {K}_d\right)}^2\right. - 4{E}_t{D}_t\Big){}^{0.5} $$

Where *E*_*t*_, *D*_*t*_, and [ED] represent the concentration of total Mg^2+^ or Zn^2+^, total dNTP, and Mg^2+^ or Zn^2+^ bound to the dNTPs, respectively. The equilibrium dissociation constant (*K*_*d*_) for dNTP with Mg^2+^ as well as Zn^2+^, Co^2+^ and Mn^2+^ was assumed to be the same as that of ATP with Mg^2+^, (*K*_*d*_ = 89.1 x 10^−6^ M) [68]. This assumption leads to an approximate value for the free concentration of these cations in reactions.

### Mismatched primer extension assays

The approach used for these assays was based on previous results [69]. The template, 5'-GGGCGAATTTAG(G/C)TTTTGTTCCCTTTAGTGAGGGTTAATTTCGAGCTTGG-3', used in these assays was a modified version of the template originally described in [70]. The underlined nts in parentheses indicate that templates with either a G or C at this position were used. The DNA primer (5′-TAACCCTCACTAAAGGGAACAAAAX-3′) used in the assays was 5′ radiolabeled and hybridized to the template at a 1:1 ratio. The ′X′ at the 3′ end of the primer denotes either G, A, T, or C (see Figure 3). Matched or mismatched primer templates (14 nM final) were incubated for 3 min at 37°C in 10.5 ul of buffer containing 50 mM Tris–HCl, 1 mM dithiothreitol, 80 mM KCl with either 2 mM MgCl_2_ or 0.4 mM ZnCl_2_ and increasing concentrations of the next correct dNTP substrate (dCTP for this template). The reaction pH was 7.7. Reactions were initiated by adding 2 μl of HIV RT (final concentration of 2 nM for Mg^2+^ reactions and 8 nM for Zn^2+^ reactions) and terminated by adding 2X loading buffer. All reactions involving matched primer-templates were carried out for 2 min with 2 mM Mg^2+^ and for 30 min with 0.4 mM Zn^2+^. Reactions with mismatched primer-templates at 2 mM Mg^2+^ or 0.4 mM Zn^2+^ were carried out for 5 min and 30 min respectively. The reactions were then electrophoresed on 16% denaturing polyacrylamide gels, dried, and imaged using a Fujifilm FLA5100 phosphoimager. Steady-state kinetic parameters *K*_*m*_, and *V*_*max*_ were then calculated as described below. The amount of free cation in each reaction was adjusted according to the dNTP concentration as described above.

### Velocity measurements and calculation of ***V***_***max***_ and ***K***_***m***_ for steady-state assays

Velocity measurement and calculation of *V*_*max*_ and *K*_*m*_ were performed as described previously for mismatch extension [69] and running-start assays [67].

### Calculation of extension rates for RNA-directed DNA synthesis in the PCR-based fidelity assay

Extension rate determinations for DNA synthesis on the 760 nt RNA template for various cations were performed as described previously [20]. Briefly, the maximal extension rate was determined by calculating the length of the longest product on 8% polyacrylamide-urea gels (19:1 acrylamide:bis-acrylamide) in reactions that had not proceeded to the end of the template (*l*). The primer length was then subtracted (20 nt) and the maximum extension rate was calculated using the formula: $\frac{\left(l-20\right)}{t}$ ,where t is the reaction time in seconds. The average extension rate was calculated by taking into account the length and the relative intensity of all extension products in a time point. Average extension rate was estimated by calculating the size in nts of each band on the gel (*s*) and subtracting the primer length (20 nt), then using the imager to determine the relative proportion (with the total being set to 1) of the total extended primers to which each band corresponded (*y*). The band’s contribution to the average extension rate can be represented by the following equation: (*s* − 20) * *y*. The average extension rate can then be calculated using the following expression:

$$ \frac{\Sigma\ \left[\left(s-20\right)*y\right]}{t} $$

Where t is the reaction time in seconds. Time points in which none of the extension products had reached the end of the template were chosen for the calculation of the maximal and the average extension rate. For example, time points of 30 s or 1 min were chosen for 0.4 mM Mn^2+^ and 0.4 mM Zn^2+^ reactions (see Figure 1).

## Abbreviations

RT: Reverse transcriptase
HIV: Human immunodeficiency virus
RNase H: Ribonuclease H
nt: nucleotide
CMF: Colony mutant frequency
